# Plaques Formed by Mutagenized Viral Populations Have Elevated Coinfection Frequencies

**DOI:** 10.1128/mBio.02020-16

**Published:** 2017-03-14

**Authors:** Elizabeth R. Aguilera, Andrea K. Erickson, Palmy R. Jesudhasan, Christopher M. Robinson, Julie K. Pfeiffer

**Affiliations:** Department of Microbiology, University of Texas Southwestern Medical Center, Dallas, Texas, USA; Vanderbilt University Medical Center

**Keywords:** coinfection, evolution, mutagen, poliovirus

## Abstract

The plaque assay is a common technique used to measure virus concentrations and is based upon the principle that each plaque represents a single infectious unit. As such, the number of plaques is expected to correlate linearly with the virus dilution plated, and each plaque should be formed by a single founder virus. Here, we examined whether more than one virus can contribute to plaque formation. By using genetic and phenotypic assays with genetically marked polioviruses, we found that multiple parental viruses are present in 5 to 7% of plaques, even at an extremely low multiplicity of infection. We demonstrated through visual and biophysical assays that, like many viral stocks, our viral stocks contain both single particles and aggregates. These data suggest that aggregated virions are capable of inducing coinfection and chimeric plaque formation. In fact, inducing virion aggregation via exposure to low pH increased coinfection in a flow cytometry-based assay. We hypothesized that plaques generated by viruses with high mutation loads may have higher coinfection frequencies due to processes restoring fitness, such as complementation and recombination. Indeed, we found that coinfection frequency correlated with mutation load, with 17% chimeric plaque formation for heavily mutagenized viruses. Importantly, the frequency of chimeric plaques may be underestimated by up to threefold, since coinfection with the same parental virus cannot be scored in our assay. This work indicates that more than one virus can contribute to plaque formation and that coinfection may assist plaque formation in situations where the amount of genome damage is high.

## IMPORTANCE

One of the most common methods to quantify viruses is the plaque assay, where it is generally presumed that each plaque represents a single infectious virus. Using genetically marked polioviruses, we demonstrate that a plaque can contain more than one parental virus, likely due to aggregates within virus stocks that induce coinfection of a cell. A relatively small number of plaques are the products of coinfection for our standard virus stocks. However, mutagenized virus stocks with increased genome damage give rise to a higher amount of plaques that are chimeric. These results suggest that coinfection may aid plaque formation of viruses with genome damage, possibly due to processes such as complementation and recombination. Overall, our results suggest that the relationship between viral dilution and plaque number may not be linear, particularly for mutagenized viral populations.

## INTRODUCTION

Viral concentrations are frequently determined using the plaque assay, which is based on the principle that each plaque represents one infectious unit (1). Many mammalian viruses have high particle-to-PFU ratios due to various factors such as assembly defects, mutations, and inefficient steps during the viral replication cycle (2–4). For most mammalian viruses, the number of plaques is directly proportional to the concentration of virus. This indicates that one infectious particle gives rise to one plaque, providing a “one-hit” model for plaque formation. However, certain viruses of plants and fungi have a “two-hit” model, whereby two particles containing different genome segments are required to coinfect the same cell to facilitate productive infection and plaque formation (5, 6). Recently, Ladner et al. reported that a mammalian virus has a “three-hit” model (7). This virus is composed of five genome segments, each packaged in five separate viral particles; however, a minimum of three segments were required to facilitate productive infection. While previous work indicates that, for most mammalian viruses, the number of plaques appears to correlate linearly with the dilution of virus plated, it is possible that some plaques may be the products of coinfection.

Several mechanisms could facilitate coinfection of viruses. Previous reports demonstrated that cells may be infected by more than one virus at a higher frequency than predicted by Poisson distribution (8–12). For example, poliovirus can be packaged in phosphatidylserine vesicles, which promotes coinfection of neighboring cells (8). Multiple coxsackievirus B3 and hepatitis A virus virions can also be packaged in vesicle-like structures (13, 14). Vesicular stomatitis virus was found to form plaques containing two different viral genomes, indicating that coinfection occurred (9). Additionally, many different viruses aggregate in solution and could induce coinfection (15–18). Viral stocks of vaccinia virus, influenza virus, adenovirus, herpesvirus, and echovirus contained virion aggregates that were resistant to antibody-mediated neutralization and/or radiation (18, 19). Poliovirus and reovirus particles can aggregate in sewage, which may contribute to initial infection of the host, and it is possible that virion aggregates exist *in vivo* (20, 21).

RNA viruses undergo error-prone replication due to lack of proofreading activity of their RNA-dependent RNA polymerase (RdRp). For poliovirus, the mutation frequency is ~10^−4^ per nucleotide per replication cycle (22). Mutations can be beneficial to viruses in some circumstances, for example by conferring resistance to neutralizing antibodies. However, most mutations are deleterious and reduce viral fitness (23). Much has been learned about RNA virus mutation-associated fitness effects from viral populations harboring increased or decreased mutation frequencies. Crotty et al. demonstrated that the nucleoside analog ribavirin (RBV) is an RNA virus mutagen. RBV is incorporated into nascent RNA by the viral RdRp, which increases transition mutations and can cause “error catastrophe” (24). Conversely, poliovirus passaged in the presence of RBV acquired a single point mutation in the RdRp, G64S, which increased fidelity of viral RNA synthesis and reduced the frequency of error in the viral population (25, 26). Importantly, poliovirus with the G64S point mutation had reduced viral fitness during infection of mice, indicating that viral population diversity is necessary for virulence (27, 28).

RNA viruses may overcome mutation-induced fitness costs by several genetic mechanisms (23). First, deleterious mutations may revert via error-prone RNA synthesis. Second, genetic recombination can generate progeny genomes lacking deleterious mutations. Recombination can occur when two viruses coinfect the same cell and exchange genetic information, likely through a copy choice mechanism (29). Recombination of genomes has been observed in poliovirus and other enteroviruses (30–36). Furthermore, defective RNA genomes are capable of undergoing recombination *in vivo*, thus restoring their fitness (36). Third, fitness may be restored by complementation, whereby viruses with distinct genetic defects complement one another. Complementation has been observed within brain tissues of poliovirus-infected mice (28). Fourth, reassortment can occur when two distinct segmented viruses coinfect the same cell and generate progeny with genome segments from both viruses. Unlike reversion, fitness restoration by recombination, complementation, and reassortment generally requires synchronous coinfection of a cell with more than one virus. Overall, these genetic processes can alter viral diversity and increase fitness.

In this work, we examined whether viral plaques are derived from a single founder and whether viruses with increased genome damage may be more reliant on coinfection for plaque formation. Through the use of a genetic assay with 10 distinct polioviruses and a phenotypic assay with 2 distinct polioviruses, we have shown that multiple parental viruses can be found within a single plaque, which we refer to as a chimeric plaque. We determined that 5 to 7% of plaques were derived from two or more viruses. To assess factors contributing to coinfection, we used dynamic light scattering and electron microscopy to demonstrate that viral stocks contain both single particles and aggregates, suggesting that infection with virion aggregates is likely responsible for chimeric plaque formation. Indeed, inducing virion aggregation via exposure to low pH increased coinfection frequency in a separate flow cytometry-based assay. We examined whether there were situations where coinfection frequencies varied and whether coinfection may assist plaque formation. Using high-fidelity G64S polioviruses that harbor fewer mutations than wild-type (WT) and RBV-mutagenized polioviruses that harbor more mutations than WT, we found that coinfection frequency correlated with mutation load. In fact, 17% of plaques from mutagenized-virus infections were the product of coinfection. This work indicates that more than one virus can contribute to plaque formation and that coinfection may assist plaque formation in situations where the amount of genome damage is high.

## RESULTS

### A small percentage of plaques are derived from more than one parental virus.

**FIG 1:**
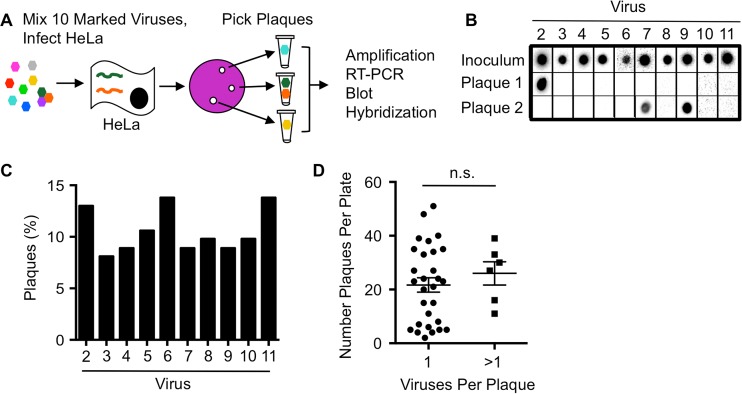
Genotypic assay reveals coinfection of polioviruses. (A) Schematic of assay design. HeLa cells were infected at an MOI of ~0.00001 with a mixture of equal amounts of 10 genetically marked viruses. Hypothetical genomes are depicted in a HeLa cell. Plaques were picked from the agar overlays after incubation at 37°C for 48 h. Plaque-forming viruses were amplified by infecting new cells and RT-PCR products were blotted and probed on a membrane to identify the virus(es) present. (B) Representative plaque virus samples detected by probes. The number of viruses present within each plaque was quantified. Plaques were scored as having a single parent virus (Plaque 1) or more than one parent virus (Plaque 2). (C) Distribution of the 10 marked viruses among all plaques. (D) Frequency of coinfected or non-coinfected plaque viruses versus the number of plaques per plate, with the mean ± standard error of the mean (error bar) shown (the difference was not significant [n.s.] as determined by Student’s *t* test).

To determine whether plaques are the product of more than one founding virus, distinct parental viruses that can be discriminated by genotype or phenotype are required. We began by using 10 genetically distinct polioviruses that each contain unique silent point mutations and are discriminated by hybridization of reverse transcription-PCR (RT-PCR) products with specific probes. We previously demonstrated that these 10 marked viruses are equally fit (37–39). We mixed equal amounts of the 10 viruses and infected HeLa cells at an extremely low multiplicity of infection (MOI) such that ~2 to 20 plaques would be generated on each plate of 10^6^ cells (Fig. 1A). Plaques were picked, viruses were amplified for a single cycle in HeLa cells, and the presence of each virus was determined by probe-specific hybridization of RT-PCR products (37–39). Plaques were scored as having a single parent virus (e.g., Plaque 1) or more than one parent virus (e.g., Plaque 2) (Fig. 1B). We examined whether each of the 10 viruses were equally represented in plaques, since skewed ratios of the input viruses could impact the observed frequency of coinfection. The distribution of viruses present within all plaques tested was reasonably even, with each of the 10 viruses nearly equally represented (Fig. 1C). Of 123 total plaques analyzed, 6 had more than one founding parental virus, and 117 had a single virus (Table 1). Therefore, 4.9% of plaques were derived from more than one parental virus.

**TABLE 1:** Frequency of plaques with more than one founding/parental virus

System	Fraction of plaques with >1 virus	% of plaques with >1 virus
10-virus genotypic assay^a^	6/123	4.9
2-virus phenotypic assay^b^	10/138	7.3

a Assay described in the legend to Fig. 1.

b Assay described in the legend to Fig. 2.

It was possible that overlapping plaques with single parental viruses were picked and incorrectly scored as chimeric. If so, the frequency of chimeric plaques should be higher on plates with more plaques present. To rule out the possibility that picking dual-parent plaques was enriched in situations where a higher number of plaques were present on the plate, we compared the number of plaques from single- and dual-parent plaques on plates (Fig. 1D). We found that dual-parent plaques were not more prevalent on plates with higher plaque numbers, indicating that cross-contamination of plaque viruses was unlikely.

Since the 10-virus genotypic assay to measure coinfection is relatively labor-intensive, we sought to simplify the screening process by using a previously characterized poliovirus mutant that can be discriminated from the WT by a phenotypic assay (29) (Fig. 2A). *3NC-202guaR* poliovirus has mutations that confer resistance to guanidine hydrochloride and temperature sensitivity (called Drug^R^/Temp^S^ hereafter). Guanidine is used as a protein denaturant, but it can also specifically inhibit poliovirus RNA synthesis (40). Conversely, WT poliovirus is guanidine hydrochloride sensitive and temperature resistant (called Drug^S^/Temp^R^ hereafter). We mixed equal amounts of Drug^R^/Temp^S^ and Drug^S^/Temp^R^ polioviruses and infected HeLa cells at an extremely low MOI such that ~2 to 20 plaques would be generated on each plate of 10^6^ cells. The infected cells were incubated under permissive conditions for both parental viruses (32°C without drug). Plaques were picked, and their growth phenotypes were analyzed to determine whether one or both parental viruses were present. Plaque formation at 32°C in the presence of drug indicated the presence of the Drug^R^/Temp^S^ parent. Plaque formation at 39.5°C in the absence of drug indicated the presence of the Drug^S^/Temp^R^ parent. Plaque formation under both conditions indicated the presence of both parental viruses (Fig. 2C). The distribution of viruses present within plaques was reasonably even, with 44.2% and 55.8% of Drug^S^/Temp^R^ and Drug^R^/Temp^S^ parental viruses, respectively (Fig. 2C). By this assay, we found that 10 out of 138 (7.3%) plaques had more than one parental virus present (Table 1). Therefore, both the genotypic and phenotypic assays indicate that ~5 to 7% of plaques that arise following infection of HeLa cells contain more than one distinct parental virus.

**FIG 2:**
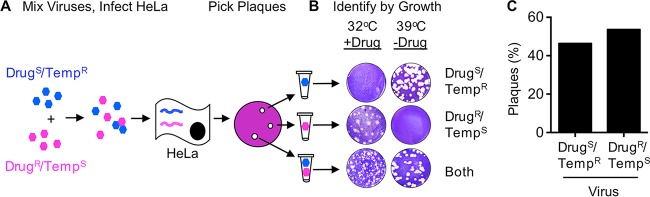
Phenotypic assay reveals coinfection of polioviruses. (A) Schematic of coinfection assay using Drug^S^/Temp^R^ and Drug^R^/Temp^S^ viruses. The two parental viruses were mixed, the cells were incubated, and HeLa cells were infected with the viral mixture at an MOI of ~0.00001. Hypothetical viral genomes are depicted in a HeLa cell. Plaques were picked 4 or 5 days after adding agar overlay at 32°C in the absence of guanidine (permissive conditions). (B) Representative plaques in the phenotypic scoring assay. Plaque-forming viruses were plated on HeLa cells under dual selective conditions as indicated. (C) Distribution of the two parental viruses among all plaques.

### Poliovirus stocks contain viral aggregates.

**FIG 3:**
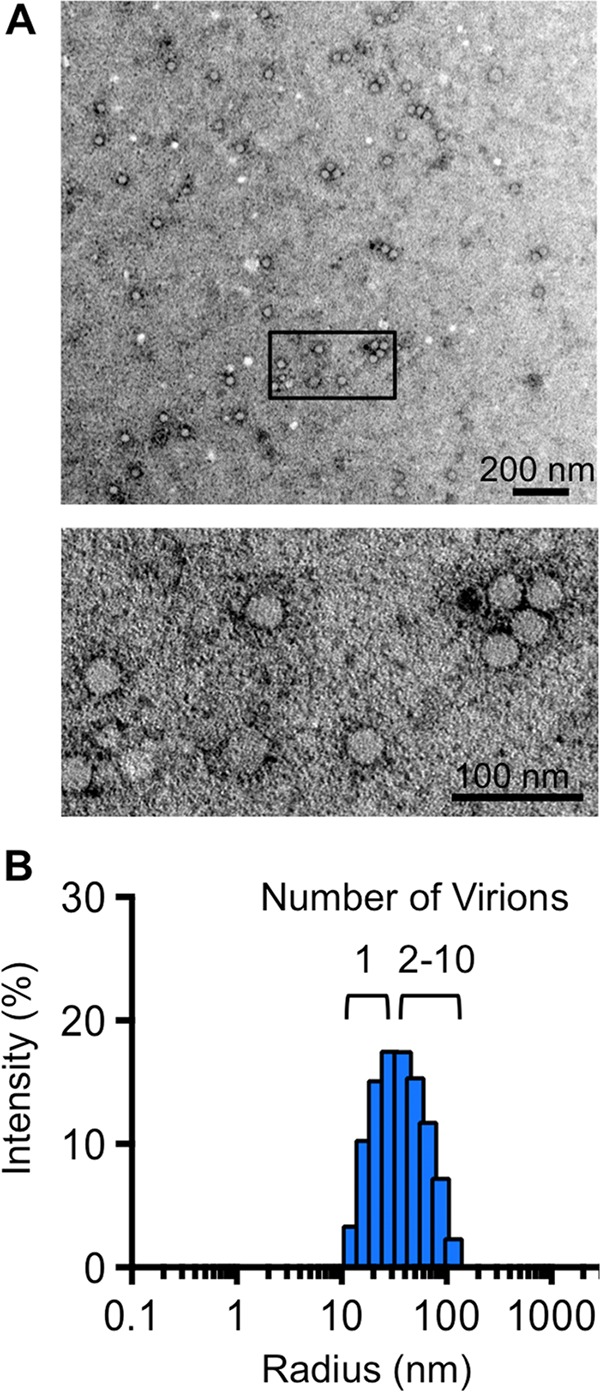
Stocks of poliovirus contain aggregates. (A) Transmission electron microscopy of a representative poliovirus stock. Viral particles were imaged at a magnification of ×13,000 (top image) or ×30,000 (bottom image [a detail of the boxed region in the top image]). (B) Dynamic light scattering analysis of a representative poliovirus stock. Virus stock was diluted to 5 × 10^4^ PFU/ml and centrifuged for 10 min prior to analysis on a Protein Solutions DynaPro instrument. The poliovirus radius is 15 nm.

To examine whether coinfection of poliovirus may be due to viral aggregation, we examined representative viral stocks using visual and biophysical assays. We first examined viruses using electron microscopy and observed single viral particles and aggregated viral particles (Fig. 3A), in agreement with previous studies (15–17). To quantify poliovirus aggregation, we used dynamic light scattering, which measures the size of particles in solution. Using this assay, we observed that our viral stock contained both single particles (15 nm radius) and aggregates ranging from 2 to 10 particles (Fig. 3B). Overall, these results indicate that viral stocks contain aggregates, and we speculated that virion aggregation facilitates coinfection of viruses.

### Inducing aggregation increases coinfection frequency.

**FIG 4:**
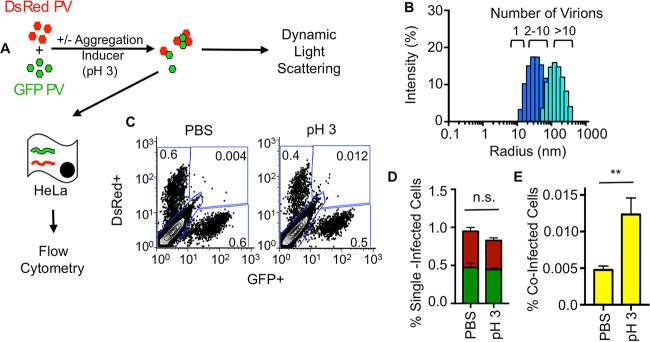
Flow cytometry-based assay demonstrates correlation between aggregation and coinfection. (A) Schematic of flow cytometry-based assay. GFP- and DsRed-expressing polioviruses were mixed in the presence or absence of aggregation-inducing conditions (with or without exposure to pH 3 solution for 4 h) prior to analysis by dynamic light scattering or infection of HeLa cells at an MOI of 0.01. At 16 hpi, infection was quantified using flow cytometry. (B) Dynamic light-scattering analysis of viruses exposed to PBS (royal blue data are the same data shown in Fig. 3B) or viruses exposed to glycine-HCl buffer at pH 3 for 4 h (turquoise). Samples were processed as described in the legend to Fig. 3. (C) Representative FACS plots showing quantification of DsRed, GFP, or dual-positive cells. The units for the *x* and *y* axes are GFP and DsRed fluorescence intensity, respectively. The numbers in each gate indicate the percentage positive of the total cell population of 2 × 10^5^ cells counted. Gates were drawn from FACS plots of HeLa cells exposed to glycine-HCl at pH 3 in the absence of PV (bottom left gate), infected with 1 × 10^4^ PFU GFP-PV (bottom right gate), infected with 1 × 10^4^ PFU DsRed-PV (top left gate) or infected with 1 × 10^4^ PFU GFP-PV and 1 × 10^4^ PFU DsRed-PV (top right gate). (D) Percentage of cells infected by single viruses (labeled with GFP or DsRed). (E) Percentage of coinfected cells, positive for both GFP and DsRed (top right gate in panel C). Results are presented as mean ± standard error of the mean (*n* = 9). Statistical significance was determined by Student’s *t* test as follows: **, *P* < 0.005; n.s., not significant.

To test the hypothesis that virion aggregation facilitates coinfection, we examined coinfection frequency for virions exposed to conditions that induce aggregation using a minimally labor-intensive flow cytometry-based assay. Polioviruses expressing either green fluorescent protein (GFP) or DsRed (41) were mixed and incubated for 4 h in phosphate-buffered saline (PBS) (control) or in glycine-HCl buffer, pH 3, which induces aggregation of poliovirus (15–17) (Fig. 4A). Viruses were then used to infect HeLa cells at an MOI of 0.01, such that ~99% of cells remain uninfected. Sixteen hours postinfection, cells were subjected to flow cytometry to quantify the percentage of uninfected, singly infected (red or green), or coinfected cells (red and green, dual positive). To ensure that the low-pH treatment induced virion aggregation, we measured particle size using dynamic light scattering. Indeed, viruses exposed to low pH had increased radii compared with viruses exposed to PBS, confirming that low pH induced virion aggregation (Fig. 4B). Based on an MOI of 0.01, approximately 1% of cells infected with PBS-treated viruses were infected with a single virus (Fig. 4C). However, 0.83% of cells infected with low-pH-treated viruses were infected with a single virus, suggesting that virion aggregation slightly reduced the total number of infectious units. A small percentage, 0.0048%, of cells infected with PBS-treated viruses were coinfected with both DsRed- and GFP-expressing viruses (Fig. 4D), which is close to the predicted number of coinfected cells based on the Poisson distribution (0.005%). Interestingly, coinfection was increased 2.6-fold in low-pH-treated viruses, suggesting that aggregation enhances coinfection. Furthermore, these data indicate that coinfection can occur during infections performed in liquid culture.

### Coinfection frequency correlates with mutation frequency.

**FIG 5:**
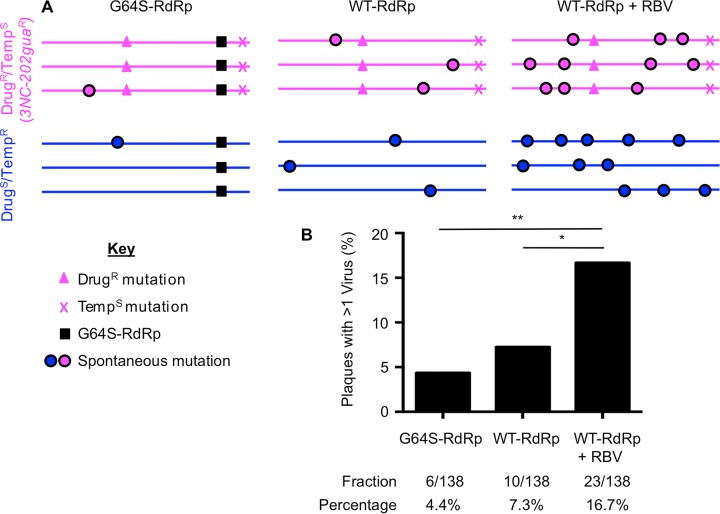
Coinfection frequency of poliovirus correlates with genome damage. (A) Schematic of viral genomes showing engineered versus representative spontaneous mutations. (B) Coinfection frequencies of high-fidelity/low-mutation viruses (G64S-RdRp), intermediate-mutation viruses (WT-RdRp), and high-mutation viruses (WT-RdRp+RBV) were performed as described for the phenotypic assay (Fig. 2). The value of coinfection for WT-RdRp virus is the same as presented in Table 1 for the phenotypic assay. Statistically significant differences were observed between WT-RdRp and WT-RdRp+RBV viruses (*, *P* = 0.0248), and between G64S-RdRp and WT-RdRp+RBV viruses (**, *P* = 0.0013) using Fisher’s exact test.

We hypothesized that coinfection would rescue plaque formation for heavily mutagenized viruses due to processes such as complementation and recombination. Conversely, we hypothesized that viruses with fewer mutations would be less reliant on coinfection for plaque formation. To test this, we compared chimeric plaque frequencies of virus stocks with low, intermediate, or high mutation frequencies (Fig. 5). We used our existing data for the Drug^S^/Temp^R^ and Drug^R^/Temp^S^ viruses containing WT RdRp (WT-RdRp viruses) as a proxy for intermediate-error frequency viral populations. To test viruses with low error frequencies, we used Drug^S^/Temp^R^ and Drug^R^/Temp^S^ viruses harboring the G64S mutation in the RdRp (G64S-RdRp viruses), which confers higher fidelity. G64S-RdRp viruses and WT-RdRp viruses were equally fit in cell culture and grew to similar titers, but G64S-RdRp viruses had 4.5-fold-fewer mutations than WT-RdRp viruses, in agreement with previous studies (Table 2) (25, 26). To test viruses with high error frequencies, we used Drug^S^/Temp^R^ and Drug^R^/Temp^S^ viruses passaged in the presence of RBV. For each virus, we infected HeLa cells in the presence of 800 μM RBV for a single cycle of replication, harvested progeny viruses, and repeated this cycle 4 or 5 times to generate mutagenized viral populations. In agreement with previous studies, these mutagenized viruses had reduced fitness, with 8.3-fold-lower titers and 21-fold-more mutations than viruses passaged in the absence of RBV (Table 2) (24). To confirm that mutagenized viral genomes had reduced specific infectivity compared with nonmutagenized WT-RdRp or G64S-RdRp viral genomes, viral RNA was extracted from 1 × 10^6^ PFU and quantified by quantitative RT-PCR (qRT-PCR). Indeed, RBV-mutagenized viruses required 2.5-fold- or 3.4-fold-more RNA to form the same number of plaques as WT-RdRP or G64S-RdRp viruses, respectively (Table 2). Therefore, these mutagenized viruses formed fewer plaques than nonmutagenized viruses due to reduced specific infectivity of their RNA genomes (Table 2) (24). We performed mixed infections for each matched set of Drug^S^/Temp^R^ and Drug^R^/Temp^S^ viruses (high fidelity [G64S-RdRp] or low fidelity [WT-RdRp+RBV]) and determined the coinfection frequency using the phenotypic assay (Fig. 2 and 5A). We observed that the coinfection frequency with G64S-RdRp viruses was decreased (3.2%) in comparison to the WT-RdRp viruses (7.3%) (Fig. 5B and Table 2). Additionally, viruses passaged in the presence of RBV had the highest percentage (16.7%) of coinfection (Fig. 5B). Overall, these data show that coinfection frequency correlates with the error frequency of the viral population, with increased coinfection among plaques generated by heavily mutagenized viruses.

**TABLE 2:** Generating virus populations with different error frequencies

Virus	Titer^a^^,^^e^	Error frequency^b^^,^^e^	No. of RNA copies/1 × 10^6^ PFU^c^^,^^e^	Specific infectivity^d^^,^^e^
G64S-RdRp	2.9 × 10^9^ (1.45×)	1.64 × 10^−5^ (0.22×)	4.5 × 10^7^ (0.7×)	2.2 × 10^−2^ (1.3×)
WT-RdRp	2.0 × 10^9^ (1×)	7.5 × 10^−5^ (1×)	6.1 × 10^7^ (1×)	1.7 × 10^−2^ (1×)
WT-RdRp+RBV	2.4 × 10^8^ (0.12×)	1.6 × 10^−3^ (21×)	1.5 × 10^8^ (2.5×)	6.6 × 10^−3^ (0.4×)

a Titer in PFU per milliliter (PFU/ml).

b The error frequency was determined by quantifying the frequency of guanidine resistance (number of PFU/ml in the presence of 1 mM guanidine hydrochloride divided by the number of PFU/ml in the absence of drug).

c Viral RNA was extracted from 1 × 10^6^ PFU of each virus and quantified by qRT-PCR.

d Specific infectivity was calculated by dividing 1 × 10^6^ PFU by the number of RNA copies.

e The numbers in parentheses are the values normalized to the value for the WT-RdRp virus.

## DISCUSSION

Coinfection of RNA viruses can promote genetic diversity and emergence of novel viruses. We found that some plaques are the result of coinfection and contain two or more parental viruses. Furthermore, we determined that coinfection frequency correlates with the level of mutation-induced genome damage. Importantly, these effects would have been masked had we not used genetically distinct viruses.

**FIG 6:**
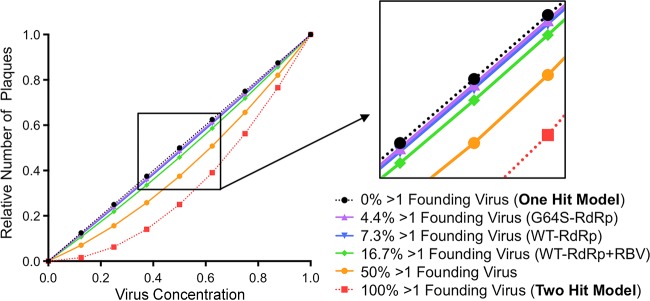
Theoretical relationship between virus dilution and plaque numbers at different coinfection frequencies. Plaque assays are based on the dose-response curve of a one-hit model (calculation depicted by the dotted black line) where each plaque is formed by one infectious unit. Certain plant and fungal viruses have two-hit kinetics (calculation depicted by the dotted red line), where two viral genomes per cell are required for productive infection and plaque formation. Purple, blue, and green lines represent calculations using data obtained in Fig. 5 for G64S-RdRp, WT-RdRp, and WT-RdRp+RBV viruses, respectively. The solid orange line represents the theoretical curve for a coinfection frequency of 50%. At low coinfection frequencies (e.g., 3.2% and 7.3%), the curvatures of the lines are minimal, and therefore, the relationship between dilution and the number of plaques is nearly linear (see the inset).

Our data show that 3 to 17% of plaques contain more than one founding virus. These data do not conform with the “one-hit” model for plaque formation whereby one infectious particle gives rise to one plaque (Fig. 6, calculation depicted by the dotted black line). Certain viruses of plant and fungi have a two-hit model, whereby two particles containing different genome segments are required to coinfect the same cell to facilitate productive infection (Fig. 6, calculation depicted by the dotted red line) (5, 6). We used our observed percentages of chimeric plaques to calculate the theoretical relationship between virus dilution and number of plaques. As shown in Fig. 6, these lines all fall between the one-hit and two-hit model lines, although all are much closer to the one-hit model line. Nonetheless, even this relatively low level of coinfection confers a slight “bend” to the one-hit model line, particularly for RBV-mutagenized viruses. These results indicate that the relationship between viral dilution plated and number of plaques is not linear, particularly for mutagenized viruses. Furthermore, even at extremely low MOIs, cells may be infected with more than one virus at a higher frequency than that predicted by the Poisson distribution.

Although the data presented here indicate that 3 to 17% of plaques arose from coinfection of two different parental viruses, the actual frequency is likely higher because only one-third of possible coinfection events are observable in our phenotypic assay. For example, coinfection with the same parental virus (e.g., Drug^S^/Temp^R^ + Drug^S^/Temp^R^ or Drug^R^/Temp^S^ + Drug^R^/Temp^S^) is scored as single-parent plaques in the phenotypic assay. Additionally, the presence of three or more parental viruses cannot be scored by the phenotypic assay. Indeed, using our genotypic assay, one plaque contained 3 of the 10 parental viruses (data not shown). Furthermore, preaggregated viruses within parental virus stocks may limit even “mixing” and reaggregation with viruses from other parental virus stocks, which could limit observable coinfection in our system. Therefore, our observed chimeric plaque frequencies (3 to 17%) are likely underestimates, and the actual frequency of plaques containing more than one parental virus could be up to three times higher. For nonmutagenized viral populations, these frequencies of chimeric plaques would still fall relatively close to the linear one-hit model line (e.g., the green line in Fig. 6), making standard plaque assay dilution series appear nearly linear and/or within a standard deviation of the assay. However, for mutagenized viral populations, the relationship between dilution plated and plaque number could become nonlinear enough to affect quantification of virus concentration. If our observed chimeric-plaque frequency is underestimated by threefold for mutagenized viruses, 50% of the plaques would be the product of coinfection, and the relationship between dilution plated and plaque number becomes obviously nonlinear (see the orange line in Fig. 6). Although our observed coinfection frequencies may be underestimates, several factors may limit productive coinfection. For example, some virions in a population are nonviable, because they lack a genome and therefore cannot productively infect or coinfect a cell. Additionally, virion aggregates in our stocks were generally composed of a small number of virions. These types of factors pose an upper limit on coinfection frequency and chimeric-plaque formation.

Our work demonstrates that plaque formation correlates with the amount of genome damage present within the viral genome, perhaps due to restoration of fitness via recombination or complementation. Given that RNA viruses have high particle-to-PFU ratios, partly because of mutations, it is possible that coinfection-mediated fitness restoration could promote “viral resurrection” of defective genomes. Overall, our findings indicate that multiple virions can contribute to plaque formation.

## MATERIALS AND METHODS

### Cells and viruses.

HeLa cells were propagated in Dulbecco’s modified Eagle’s medium (DMEM) supplemented with 10% calf serum and 1% penicillin-streptomycin. All infections were performed using viruses derived from Mahoney serotype 1 poliovirus infectious cDNA clone variants containing wild-type RNA-dependent RNA polymerase (WT-RdRp) or RdRp with the G64S mutation (G64S-RdRp) with or without *3NC-202guaR* mutations (see Fig. 5A for schematic) (25, 29). The *3NC-202guaR* virus contains two mutations that confer guanidine resistance (2C-M187L and 2C-V250A) and an insertion in the 3′ noncoding region that confers temperature sensitivity (3-NC202) (29, 42). To generate highly mutagenized poliovirus, 1 × 10^6^ HeLa cells were pretreated with 800 μM ribavirin (RBV) (Sigma) for 1 h. Approximately 1 × 10^5^ PFU of virus was used to infect the cells for 30 min at 37°C and 5% CO_2_. Unattached virus was washed with phosphate-buffered saline (PBS), and medium containing 800 μM RBV was added to the cells. Virus was harvested at approximately 7 h postinfection (hpi) in 1 ml phosphate-buffered saline supplemented with 100 μg/ml CaCl_2_ and 100 μg/ml MgCl_2_ (PBS+). Passage of virus in the presence of RBV was repeated 4 or 5 times. For the genotypic assay, 10 polioviruses derived from Mahoney serotype 1, each containing unique silent point mutations in the VP3 capsid-coding region, were used as previously described (37).

### Quantifying dual-parent versus single-parent plaque viruses.

The 10 polioviruses containing unique silent point mutations were mixed in equivalent amounts with PBS+ and incubated at 37°C for 1 h. After incubation, the viral mixture was diluted to a multiplicity of infection (MOI) of ~0.00001 and plated onto 10-cm plates seeded with 1 × 10^6^ HeLa cells. After attachment, unbound virus was removed, and an agar overlay was added as previously described (25). The plates were incubated at 37°C and 5% CO_2_ for 48 h, the plaques were picked, and plaque agar plugs were placed into tubes with each tube containing 1 ml PBS+. Plaque stocks were freeze-thawed three times to release virus, and 200-μl portions of these viruses were used to infect fresh HeLa cells to generate viral RNA for analysis of parental viruses. At 6 hpi, total RNA was isolated using Tri Reagent (Sigma-Aldrich) according to the manufacturer’s instructions. cDNA synthesis, PCR, blotting, and hybridization using ^32^P-labeled oligonucleotide probes specific for each of the 10 viral sequences was performed as previously described (37).

In the phenotypic assay, 1 × 10^5^ PFU of guanidine hydrochloride-sensitive and temperature-resistant (Drug^S^/Temp^R^) poliovirus and guanidine hydrochloride-resistant and temperature-sensitive (Drug^R^/Temp^S^) poliovirus were incubated in PBS+ for 1 h at 37°C. After incubation, the viral mixture was diluted to an MOI of ~0.00001 and plated onto 10-cm plates seeded with 1 × 10^6^ HeLa cells. After attachment, unbound virus was removed, and agar overlay was added as previously described (25). The plates were incubated at 32°C and 5% CO_2_ in the absence of guanidine hydrochloride (Sigma) for 4 or 5 days. Plaques were picked and placed into tubes containing 1 ml PBS+. To release virus, plaques stocks were freeze-thawed three times prior to screening. Plaque viruses were screened by performing plaque assays under selective growth conditions (32°C with 1 mM guanidine or 39.5°C without guanidine). To ensure that phenotypes could be discriminated accurately, preliminary experiments were performed in a blind manner (researchers were blind to the trial), and Drug^S^/Temp^R^ versus Drug^R^/Temp^S^ viruses were correctly scored. Of several hundred plaques that were picked during the course of this study, 14 did not contain detectable virus and were not included in the analysis. It is likely that these “plaques” were nonviral defects in the monolayer. Because no detectable virus was present in these samples, it is unlikely that inefficient/abortive infections that yield low-level virus are common in our system.

### Analysis of poliovirus aggregation by electron microscopy.

Poliovirus stocks were purified by cesium chloride gradient centrifugation and were concentrated and desalted using Amicon filters (Millipore) as previously described (43). Poliovirus samples containing 9.3 × 10^6^ PFU were inactivated by treating with 2.5% glutaraldehyde for 1 h at room temperature. After inactivation, 2.5 µl of the inactivated virus was placed on 400-mesh carbon-coated copper grids that had been glow discharged for 30 s using PELCO EasiGlow 91000. The grids were stained with 2% phosphotungstic acid and examined using a TEI Technai G^2^ Spirit Biotwin transmission electron microscope (FEI, Hillsboro, OR) equipped with a Gatan ultrascan charge-coupled-device (CCD) camera, operated at an acceleration voltage of 120 kV. Images were taken at magnifications of ×13,000 and ×30,000.

### Quantifying poliovirus aggregation using dynamic light scattering.

Samples of 5 × 10^4^ PFU gradient-purified poliovirus (see electron microscopy methods above) were prepared in a total volume of 20 µl, and samples were centrifuged at 10,000 rpm for 10 min before data acquisition to remove dust or contaminants. Experiments were performed on a Protein Solutions DynaPro instrument equipped with a temperature-controlled microsampler (Wyatt Technology) using 20-s acquisition time and 20% laser power. Each measurement was an average of 20 data points. The data were processed with the program Dynamics V6. The radii and the size distribution were calculated with the regularization algorithm provided by the software.

### Flow cytometry-based assay for coinfection.

Viruses derived from Mahoney serotype 1 poliovirus (PV) infectious cDNA clone encoding *Aequorea coerulescens* green fluorescent protein (GFP) or *Discosoma* sp*.* red (DsRed) fluorescent proteins inserted after amino acid 144 of PV protein 2A (PV-2A144-GFP and PV-2A144-DsRed) have been previously described (41). Equal amounts of GFP-labeled PV (GFP-PV) and DsRed-labeled PV (DsRed-PV) (1 × 10^4^ PFU each) were incubated in 200 μl of PBS (pH 7.4) or 0.05 M glycine hydrochloride (glycine HCl)–H_2_O buffer (pH 3) for 4 h at room temperature. HeLa cells grown in six-well plates containing approximately 2 × 10^6^ cells/well were mock infected with pH 3 buffer or infected with the GFP-PV and/or DsRed-PV mixtures for 15 min at 37°C. The cells were washed with PBS, and 2 ml of DMEM supplemented with 5% calf serum and 1% penicillin-streptomycin was added to each well. After incubation for 16 h at 37°C and 5% CO_2_, cells were harvested using 0.1% trypsin–0.05% EDTA solution, washed, and fixed with 2% paraformaldehyde fixation solution for 15 min at room temperature and resuspended in 300 μl of PBS containing 2% fetal bovine serum. Expression of GFP and DsRed was determined using a FACSCalibur cytometer equipped with 488- and 635-nm lasers. Fluorescence-activated cell sorting (FACS) data were analyzed using FlowJo software. Given the low MOI, a relatively large number of cells (2 × 10^5^) were counted for each experimental condition. Experiments performed with a range of MOIs demonstrated that the conditions used here were in the linear range and above the detection limit (data not shown). Additionally, the observed coinfection frequency of 0.0048% is nearly identical to the coinfection frequency predicted by the Poisson distribution (0.005%) (Fig. 4E). Gates were determined using uninfected cells or singly infected cells as indicated in the legend to Fig. 4.

### Quantifying the mutation frequencies of viruses.

Error frequencies were determined by acquisition of guanidine resistance, as previously described (25). Because the Drug^R^/Temp^S^ viruses are uninformative for this assay, we scored the frequency of guanidine resistance in the Drug^S^/Temp^R^ viruses (grown in parallel with the Drug^R^/Temp^S^ viruses) in the (i) WT-RdRp background, (ii) G64S-RdRp background, or (iii) WT-RdRp background mutagenized with RBV (see Fig. 4A for a schematic). Viral dilutions were plated on approximately 1 × 10^6^ HeLa cells to determine the viral titer by plaque assay at 32°C and 39.5°C in the presence or absence of 1 mM guanidine hydrochloride. The error frequencies were determined by dividing the number of PFU per milliliter obtained in the presence of drug by the number of PFU per milliliter in the absence of drug. Note that the highest observed error frequency (Drug^R^ reversion frequency) was 1.6 × 10^−3^, meaning that 1 in every 625 viruses lost the guanidine resistance phenotype. Because the number of plaques screened in the phenotypic assay is much lower than this reversion frequency, it is unlikely that gain or loss of the Drug^R^ marker impacted quantification of coinfection. To quantify the relative specific infectivity for WT-RdRp, G64S-RdRp, and WT-RdRp+RBV stocks, RNA was extracted from 1 × 10^6^ PFU of each stock using Tri Reagent (Sigma) with carrier RNA from 10^6^ HeLa cells, and quantification of poliovirus RNA was performed using quantitative reverse transcription-PCR (RT-PCR). Reverse transcription was performed with Superscript II (Invitrogen) using an antisense primer, 5′ TGTAACGCCTCCAAATTCCAC 3′, in the VP2 capsid-coding region. To perform quantitative PCR (qPCR), 5 μl of the cDNA reaction mixture was mixed with SYBR green PCR master mix reagent (Applied Biosystems) and a 10 μM concentration of each primer. The VP2 capsid region was amplified with the sense primer 5′ TGAGGGACATGGGACTCTTT 3′ and the antisense primer above using an Applied Biosystems 7500 system. Cycling conditions were 1 cycle for 2 min at 50°C and 10 min at 95°C, followed by 40 cycles, with 1 cycle consisting of 15 s at 95°C and 1 min at 60°C. The qPCRs were performed in duplicate from two independent RNA preparations and quantified using a standard curve generated with poliovirus plasmid DNA samples. Analysis of standard curve and data were determined as previously described (44, 45). Specific infectivity was determined by dividing 1 × 10^6^ PFU by the relative amount of RNA (Table 2).

### Relationship between virus dilution and plaque numbers at different coinfection frequencies.

To generate the graph shown in Fig. 6, we used the following formula to calculate the predicted number of plaques generated by several dilutions of virus over a range of coinfection frequencies: [(dilution)^1 ^× fraction with one virus] + [(dilution)^2^ × fraction with two viruses] = number of plaques.

## References

[1] Dulbecco R, Vogt M 1953 Some problems of animal virology as studied by the plaque technique. Cold Spring Harb Symp Quant Biol 18:273–279. doi:10.1101/SQB.1953.018.01.039. 13168995

[2] Muller HJ 1964 The relation of recombination to mutational advance. Mutat Res 106:2–9. doi:10.1016/0027-5107(64)90047-8. 14195748

[3] Duarte E, Clarke D, Moya A, Domingo E, Holland J 1992 Rapid fitness losses in mammalian RNA virus clones due to Muller’s ratchet. Proc Natl Acad Sci U S A 89:6015–6019. doi:10.1073/pnas.89.13.6015. 1321432 PMC402129

[4] Yuste E, Sánchez-Palomino S, Casado C, Domingo E, López-Galíndez C 1999 Drastic fitness loss in human immunodeficiency virus type 1 upon serial bottleneck events. J Virol 73:2745–2751. 10074121 10.1128/jvi.73.4.2745-2751.1999 PMC104031

[5] Ghabrial SA, Suzuki N 2009 Viruses of plant pathogenic fungi. Annu Rev Phytopathol 47:353–384. doi:10.1146/annurev-phyto-080508-081932. 19400634

[6] Rao AL 2006 Genome packaging by spherical plant RNA viruses. Annu Rev Phytopathol 44:61–87. doi:10.1146/annurev.phyto.44.070505.143334. 16480335

[7] Ladner JT, Wiley MR, Beitzel B, Auguste AJ, Dupuis AP II, Lindquist ME, Sibley SD, Kota KP, Fetterer D, Eastwood G, Kimmel D, Prieto K, Guzman H, Aliota MT, Reyes D, Brueggemann EE, St John L, Hyeroba D, Lauck M, Friedrich TC, O’Connor DH, Gestole MC, Cazares LH, Popov VL, Castro-Llanos F, Kochel TJ, Kenny T, White B, Ward MD, Loaiza JR, Goldberg TL, Weaver SC, Kramer LD, Tesh RB, Palacios G 2016 A multicomponent animal virus isolated from mosquitoes. Cell Host Microbe 20:357–367. doi:10.1016/j.chom.2016.07.011. 27569558 PMC5025392

[8] Chen YH, Du W, Hagemeijer MC, Takvorian PM, Pau C, Cali A, Brantner CA, Stempinski ES, Connelly PS, Ma HC, Jiang P, Wimmer E, Altan-Bonnet G, Altan-Bonnet N 2015 Phosphatidylserine vesicles enable efficient en bloc transmission of enteroviruses. Cell 160:619–630. doi:10.1016/j.cell.2015.01.032. 25679758 PMC6704014

[9] Combe M, Garijo R, Geller R, Cuevas JM, Sanjuán R 2015 Single-cell analysis of RNA virus infection identifies multiple genetically diverse viral genomes within single infectious units. Cell Host Microbe 18:424–432. doi:10.1016/j.chom.2015.09.009. 26468746 PMC4617633

[10] Bird SW, Kirkegaard K 2015 Escape of non-enveloped virus from intact cells. Virology 479-480:444–449. doi:10.1016/j.virol.2015.03.044. 25890822 PMC4440412

[11] Bird SW, Maynard ND, Covert MW, Kirkegaard K 2014 Nonlytic viral spread enhanced by autophagy components. Proc Natl Acad Sci U S A 111:13081–13086. doi:10.1073/pnas.1401437111. 25157142 PMC4246951

[12] Shabram P, Aguilar-Cordova E 2000 Multiplicity of infection/multiplicity of confusion. Mol Ther 2:420–421. doi:10.1006/mthe.2000.0212. 11082315

[13] Feng Z, Hensley L, McKnight KL, Hu F, Madden V, Ping L, Jeong SH, Walker C, Lanford RE, Lemon SM 2013 A pathogenic picornavirus acquires an envelope by hijacking cellular membranes. Nature 496:367–371. doi:10.1038/nature12029. 23542590 PMC3631468

[14] Robinson SM, Tsueng G, Sin J, Mangale V, Rahawi S, McIntyre LL, Williams W, Kha N, Cruz C, Hancock BM, Nguyen DP, Sayen MR, Hilton BJ, Doran KS, Segall AM, Wolkowicz R, Cornell CT, Whitton JL, Gottlieb RA, Feuer R 2014 Coxsackievirus B exits the host cell in shed microvesicles displaying autophagosomal markers. PLoS Pathog 10:e1004045. doi:10.1371/journal.ppat.1004045. 24722773 PMC3983045

[15] Floyd R, Sharp DG 1977 Aggregation of poliovirus and reovirus by dilution in water. Appl Environ Microbiol 33:159–167. 13711 10.1128/aem.33.1.159-167.1977 PMC170617

[16] Floyd R, Sharp DG 1978 Viral aggregation: effects of salts on the aggregation of poliovirus and reovirus at low pH. Appl Environ Microbiol 35:1084–1094. 28078 10.1128/aem.35.6.1084-1094.1978 PMC242989

[17] Floyd R, Sharp DG 1979 Viral aggregation: buffer effects in the aggregation of poliovirus and reovirus at low and high pH. Appl Environ Microbiol 38:395–401. 43706 10.1128/aem.38.3.395-401.1979 PMC243506

[18] Wallis C, Melnick JL 1967 Virus aggregation as the cause of the non-neutralizable persistent fraction. J Virol 1:478–488. 4318956 10.1128/jvi.1.3.478-488.1967 PMC375261

[19] Sharp DG, Dunlap RC 1966 Multiplicity reactivation of vaccinia virus in the cells of the chorioallantoic membrane. Exp Biol Med 123:111–114. doi:10.3181/00379727-123-31416. 5924408

[20] Young DC, Sharp DG 1977 Poliovirus aggregates and their survival in water. Appl Environ Microbiol 33:168–177. 189686 10.1128/aem.33.1.168-177.1977 PMC170618

[21] Sharp DG, Floyd R, Johnson JD 1975 Nature of the surviving plaque-forming unit of reovirus in water containing bromine. Appl Microbiol 29:94–101. 1167388 10.1128/am.29.1.94-101.1975 PMC186917

[22] Ward CD, Stokes MA, Flanegan JB 1988 Direct measurement of the poliovirus RNA polymerase error frequency in vitro. J Virol 62:558–562. 2826815 10.1128/jvi.62.2.558-562.1988 PMC250568

[23] Domingo E, Holland JJ 1997 RNA virus mutations and fitness for survival. Annu Rev Microbiol 51:151–178. doi:10.1146/annurev.micro.51.1.151. 9343347

[24] Crotty S, Cameron CE, Andino R 2001 RNA virus error catastrophe: direct molecular test by using ribavirin. Proc Natl Acad Sci U S A 98:6895–6900. doi:10.1073/pnas.111085598. 11371613 PMC34449

[25] Pfeiffer JK, Kirkegaard K 2003 A single mutation in poliovirus RNA-dependent RNA polymerase confers resistance to mutagenic nucleotide analogs via increased fidelity. Proc Natl Acad Sci U S A 100:7289–7294. doi:10.1073/pnas.1232294100. 12754380 PMC165868

[26] Arnold JJ, Vignuzzi M, Stone JK, Andino R, Cameron CE 2005 Remote site control of an active site fidelity checkpoint in a viral RNA-dependent RNA polymerase. J Biol Chem 280:25706–25716. doi:10.1074/jbc.M503444200. 15878882 PMC1557591

[27] Pfeiffer JK, Kirkegaard K 2005 Increased fidelity reduces poliovirus fitness and virulence under selective pressure in mice. PLoS Pathog 1:e11. doi:10.1371/journal.ppat.0010011. 16220146 PMC1250929

[28] Vignuzzi M, Stone JK, Andino R 2005 Ribavirin and lethal mutagenesis of poliovirus: molecular mechanisms, resistance and biological implications. Virus Res 107:173–181. doi:10.1016/j.virusres.2004.11.007. 15649563

[29] Kirkegaard K, Baltimore D 1986 The mechanism of RNA recombination in poliovirus. Cell 47:433–443. doi:10.1016/0092-8674(86)90600-8. 3021340 PMC7133339

[30] Sergiescu D, Aubert-Combiescu A, Crainic R 1969 Recombination between guanidine-resistant and dextran sulfate-resistant mutants of type 1 poliovirus. J Virol 3:326–330. 4305674 10.1128/jvi.3.3.326-330.1969 PMC375771

[31] Furione M, Guillot S, Otelea D, Balanant J, Candrea A, Crainic R 1993 Polioviruses with natural recombinant genomes isolated from vaccine-associated paralytic poliomyelitis. Virology 196:199–208. doi:10.1006/viro.1993.1468. 8102826

[32] Cuervo NS, Guillot S, Romanenkova N, Combiescu M, Aubert-Combiescu A, Seghier M, Caro V, Crainic R, Delpeyroux F 2001 Genomic features of intertypic recombinant Sabin poliovirus strains excreted by primary vaccinees. J Virol 75:5740–5751. doi:10.1128/JVI.75.13.5740-5751.2001. 11390576 PMC114290

[33] Dahourou G, Guillot S, Le Gall O, Crainic R 2002 Genetic recombination in wild-type poliovirus. J Gen Virol 83:3103–3110. doi:10.1099/0022-1317-83-12-3103. 12466487

[34] Arita M, Zhu SL, Yoshida H, Yoneyama T, Miyamura T, Shimizu H 2005 A Sabin 3-derived poliovirus recombinant contained a sequence homologous with indigenous human enterovirus species C in the viral polymerase coding region. J Virol 79:12650–12657. doi:10.1128/JVI.79.20.12650-12657.2005. 16188967 PMC1235834

[35] Simmonds P, Bukh J, Combet C, Deléage G, Enomoto N, Feinstone S, Halfon P, Inchauspé G, Kuiken C, Maertens G, Mizokami M, Murphy DG, Okamoto H, Pawlotsky JM, Penin F, Sablon E, Shin IT, Stuyver LJ, Thiel HJ, Viazov S, Weiner AJ, Widell A 2005 Consensus proposals for a unified system of nomenclature of hepatitis C virus genotypes. Hepatology 42:962–973. doi:10.1002/hep.20819. 16149085

[36] Holmblat B, Jégouic S, Muslin C, Blondel B, Joffret ML, Delpeyroux F 2014 Nonhomologous recombination between defective poliovirus and coxsackievirus genomes suggests a new model of genetic plasticity for picornaviruses. mBio 5:e01119-14. doi:10.1128/mBio.01119-14. 25096874 PMC4128350

[37] Kuss SK, Etheredge CA, Pfeiffer JK 2008 Multiple host barriers restrict poliovirus trafficking in mice. PLoS Pathog 4:e1000082. doi:10.1371/journal.ppat.1000082. 18535656 PMC2390757

[38] Lancaster KZ, Pfeiffer JK 2010 Limited trafficking of a neurotropic virus through inefficient retrograde axonal transport and the type I interferon response. PLoS Pathog 6:e1000791. doi:10.1371/journal.ppat.1000791. 20221252 PMC2832671

[39] Luethy LN, Erickson AK, Jesudhasan PR, Ikizler M, Dermody TS, Pfeiffer JK 2016 Comparison of three neurotropic viruses reveals differences in viral dissemination to the central nervous system. Virology 487:1–10. doi:10.1016/j.virol.2015.09.019. 26479325 PMC4679581

[40] Tershak DR 1982 Inhibition of poliovirus polymerase by guanidine in vitro. J Virol 41:313–318. 6283124 10.1128/jvi.41.1.313-318.1982 PMC256753

[41] Teterina NL, Levenson EA, Ehrenfeld E 2010 Viable polioviruses that encode 2A proteins with fluorescent protein tags. J Virol 84:1477–1488. doi:10.1128/JVI.01578-09. 19939919 PMC2812313

[42] Sarnow P, Bernstein HD, Baltimore D 1986 A poliovirus temperature-sensitive RNA synthesis mutant located in a noncoding region of the genome. Proc Natl Acad Sci U S A 83:571–575. doi:10.1073/pnas.83.3.571. 3003739 PMC322905

[43] Kuss SK, Best GT, Etheredge CA, Pruijssers AJ, Frierson JM, Hooper LV, Dermody TS, Pfeiffer JK 2011 Intestinal microbiota promote enteric virus replication and systemic pathogenesis. Science 334:249–252. doi:10.1126/science.1211057. 21998395 PMC3222156

[44] Ibarra KD, Pfeiffer JK 2009 Reduced ribavirin antiviral efficacy via nucleoside transporter-mediated drug resistance. J Virol 83:4538–4547. doi:10.1128/JVI.02280-08. 19244331 PMC2668478

[45] Bookout AL, Cummins CL, Mangelsdorf DJ, Pesola JM, Kramer MF 2006 High-throughput real-time quantitative reverse transcription PCR. Curr Protoc Mol Biol Chapter 15:Unit 15.8. doi:10.1002/0471142727.mb1508s73. 18265376

